# Investigating the effectiveness of nanotechnologies in environmental health with an emphasis on environmental health journals

**DOI:** 10.1186/s40504-021-00116-8

**Published:** 2021-09-13

**Authors:** Zahra Aghalari, Hans-Uwe Dahms, Mika Sillanpää

**Affiliations:** 1 Faculty of Public Health, Babol University of Medical Sciences, Babol, Iran; 2 Department of Biomedical Science and Environment Biology, College of Life Science, Kaohsiung Medical University, Kaohsiung, Taiwan; 3 Research Center for Environmental Medicine, KMU - Kaohsiung Medical University, Kaohsiung, 80708 Taiwan; 4 Department of Biological and Chemical Engineering, Aarhus University, Nørrebrogade 44, 8000 Aarhus C, Denmark; 5 Faculty of Science and Technology, School of Applied Physics, University Kebangsaan Malaysia, 43600 Bangi, Selangor Malaysia; 6 School of Chemistry, Shoolini University, Solan, Himachal Pradesh 173229 India

**Keywords:** Environmental health, Nanotechnology, Journals, Articles

## Abstract

**Objective:**

The use of nanotechnologies is important to reduce environmental health problems in Iran, so the present study was conducted to determine the effectiveness of nanotechnologies in environmental health. This is a cross-sectional descriptive study for 11-year periods (2008–2018) on all articles published in three specialized journals of environmental health with emphasis on the use of nanotechnologies in various fields of environmental health (water, air, sewage, waste, food, radiation, etc).

**Results:**

In this study, 774 articles related to 114 issues of 3 specialized environmental health journals were reviewed. A review of 774 articles showed that 80 articles (10.3%) were published in the field of nanotechnologies. Out of 80 articles published in the field of nanotechnology, 66 articles (82.5%) were published on the subject of water, 9 articles (11.3%) on wastewater and 5 articles (6.2%) on air pollution. Subject review of articles showed that articles using carbon nanotubes to remove natural organic pollutants, surfactants, hydroxybenzenes, phenol, dimethyl phthalates, use of titanium dioxide nanoparticles, iron-magnesium nanoparticles for wastewater treatment, Silver nanoparticles were used to remove air pollution. The results showed that published articles on nanotechnology in the field of environmental health were few.

## Introduction

Nanotechnology has emerged from the convergence of the physical sciences, chemistry and biology and is the foundation of convergent technologies (Viswanath and Kim 2017; Song et al. 2011). Nanotechnology provides the ability to work at the atomic level and to create structures that have a completely new molecular order (Liu et al. n.d.; Durgalakshmi et al. 2019). Nanoproducts are also used as powders or very thin fibers. A nanomaterial means that its structure and the size of the crystals that are formed or their atomic organization are formed in the whole body at the nanoscale (Massawe 2013; Guerra et al. 2018). Nanotechnologies are used to make microscopic and molecular-sized devices in the pharmaceutical, environmental, and energy sciences (Ray et al. 2009; Zhang et al. 2019). Nanotechnologies can also be used effectively in environmental health. In developing countries, nanoproducts such as nanotubes, nanosensors, nanocatalysts and nanocomposites are used in environmental health to eliminate or reduce water, air, soil, wastewater and food pollutants (Seferos et al. 2007; Wu et al. 2019). The united states environmental protection agency (US EPA) reports that there are many health and environmental challenges in the twenty-first century, and nanotechnology can detecting, measuring, and removing the most important pollutants from air, water, soil, and discovering new green industrial processes that reduce waste (Kumar and Jakhmola 2007). Iran is one of the developing countries and on the other hand, the use of new technologies such as nanotechnology to reduce environmental health problems in Iran is important, so the present study aims to determine the effectiveness of nanotechnologies in environmental health with an emphasis on environmental health journals.

## Materials and methods

The present descriptive cross-sectional study (retrospective) was conducted for 11-year periods (2008–2018) among all articles published in three specialized journals of environmental health. Inclusion criteria for this study included the name of environmental health in the title of the journal, having at least four issues per year and publishing articles for at least three consecutive years. According to the inclusion criteria, three Persian language journals were reviewed: Iranian Journal of Health and Environment (IJHE), Journal of Environmental Health Engineering (JEHE), Journal of Research in Environmental Health (JREH). After visiting the site of the journals, the abstract and then the full text of the articles were reviewed. Articles focusing on the use of nanotechnologies in each of the topics of environmental health (water, air, sewage, waste, food, radiation, etc.) were included in the study. Data collection was done through a researcher-made checklist. To prepare the checklist, scientometric method was used, which is a new method for identifying scientific topics and sub-disciplines in different fields (Tirgar et al. 2019; Tirgar and Aghalari 2018). According to the content of the articles, variables such as the frequency of articles by journals and year, number of articles by topic, type of nanotechnology in each of the environmental health topics were recorded in the checklist. After collecting the data, the information of all the articles was coded and entered into Excel software. Then, for descriptive statistics, scattering and table indices were used.

## Results

In the present study, 774 articles related to 114 issues of 3 specialized journals of environmental health were reviewed. Out of 774 published articles, the highest number of articles (478 articles - 61.7%) was related to Iranian Journal of Health and Environment. The highest number of articles related to the 2 years 2015 and 2016, respectively, 112 articles (14.4%) and 111 articles (14.3%) and the lowest number of articles related to the year 2008 with a frequency of 16 articles (1.2%).

A review of 774 articles published by journals showed that 80 articles (10.3%) were published in the field of nanotechnology, with the highest number of articles in this field in 2015 with 15 articles. Out of 80 articles published in the field of nanotechnology, 66 articles (82.5%) were published about water pollution, 9 articles (11.3%) about waste water and 5 articles (6.2%) about air pollution (Table 1).

**Table 1:** Frequency of articles published in the field of nanotechnology in various health topics during 2008–2018

Years of publication	Journals											
	IJHE				JEHE				JREH			
	Number of articles	Articles related to nanotechnology in the field of water	Articles related to nanotechnology in the field of wastewater	Articles related to nanotechnology in the field of air	Number of articles	Articles related to nanotechnology in the field of water	Articles related to nanotechnology in the field of wastewater	Articles related to nanotechnology in the field of air	Number of articles	Articles related to nanotechnology in the field of water	Articles related to nanotechnology in the field of wastewater	Articles related to nanotechnology in the field of air
2008	16	1	–	–	–	–	–	–	–	–	–	–
2009	32	1	–	1	–	–	–	–	–	–	–	–
2010	47	4	1	–	–	–	–	–	–	–	–	–
2011	48	3	–	–	–	–	–	–	–	–	–	–
2012	48	6	1	1	–	–	–	–	–	–	–	–
2013	48	7	–	–	16	3	–	–	–	–	–	–
2014	48	6	1	–	32	2	–	–	–	–	–	–
2015	48	5	2	1	32	6	–	–	32	–	1	–
2016	48	4	–	1	32	3	1	–	31	–	–	–
2017	48	4	–	1	32	5	1	–	29	–	–	–
2018	48	2	–	–	32	2	1	–	28	2	–	–
Total	478	43	5	5	176	21	3	–	120	2	1	–

A thematic review of articles published in the field of nanotechnology on water pollution showed that 66 articles used carbon nanotubes to remove contaminants such as natural organic matter, surfactants, hydroxybenzene, phenol, dimethyl phthalate in water. Published articles stated that carbon nanotubes are effective as adsorbents for the removal of various types of contaminants due to their high specific surface area, small structures, hollow and flexibility, and high elasticity. Nanoparticles used to remove water pollutants include zinc oxide nanoparticles, nanoseolites, iron nanoparticles, titanium dioxide nanoparticles, nanofiltration function, colloidal silver nanoparticles, nanoparticles made from cedar leaf ash, nanoparticles of copper oxide nanoparticles, nanoparticles of copper oxide nanoparticles Calcium, modified magnetite nanoparticles, magnetic activated carbon nanocomposite-zero iron, titanium oxide-graphene oxide nanocomposites were reduced.

Nine articles published on the use of nanotechnologies in the field of wastewater were about the use of titanium dioxide nanoparticles, iron-magnesium nanoparticles, the treatment of synthetic wastewater with chitosan nanocomposites, and the use of carbon nanotubes to remove wastewater contaminants. Five articles were published on the use of nanotechnologies in the field of air pollution, regarding the use of silver nanoparticles to remove airborne fungal pollution, removal of styrene from the air stream using zinc oxide nanoparticles.

## Discussion

This study showed that out of 774 articles reviewed, 10.3% of articles were published on the subject of nanotechnologies in environmental health, which indicates the lack of research related to nanotechnology in environmental health in Iran and the attention of health scientists and new technologies. It requires, because by using nanotechnology in the field of environmental health, in addition to reducing and eliminating environmental pollutants, economic benefits are also created. For example, Schmidt’s study showed that one of the benefits of using nanotechnology in the environment, in addition to reducing energy consumption, controlling pollutants, reducing costs and increasing economic productivity (Schmidt 2009).

Of the 80 articles published on nanotechnology, 6.2% articles were on air pollution and the use of silver nanoparticles to remove airborne fungal contaminants was the removal of styrene from the air stream using zinc oxide nanoparticles. The use of nanotechnologies in air pollution issues should be given more attention, because according to research, the efficiency of nanotechnology in reducing air pollution has been proven. For example, in the results of a study by Lashkarizadeh et al., It was stated that air pollution in developed countries is reduced by the use of nanotechnology (Lashkarizadeh and Eshaghi 2017). A study by Motiee et al. evaluating the effectiveness of nanotechnology on air pollution showed that the production of nanomaterials, nanotubes, nanocomposites, nanofilters and nanoparticles are examples of the application of nanotechnology to build air monitoring systems and reduce air pollutants (Motiee and Khayat 2011). Roco investigated the effect of nanotechnologies on reducing air pollutants on solid state gas censorship and reported that nanotechnology is effective for gas monitoring systems, factory gas leak detectors, fire and toxic gas detectors, ventilation control, and breathalyzers (Roco 2011).

A thematic review of articles published in the field of nanotechnology about water pollution showed that a number of articles used carbon nanotubes to remove contaminants such as natural organic matter, surfactants, hydroxybenzene, phenol, dimethyl phthalate, in aqueous media. In recent years, carbon nanoparticles, including carbon nanotubes, have attracted the attention of scientists due to their ability to isolate contaminants such as heavy metals, radionuclides, hazardous organic and inorganic compounds (Yu et al. 2012; Das et al. 2015). Alizadeh and etal., in a study aimed at determining the applications of nanotechnology in the environment and new energies reported that carbon nanotubes are a suitable technology that can selectively absorb gas in a stream containing a mixture of gases. This capability of nanotubes can be used to remove hazardous gases from automobile gasoline as well as environmental pollutants as well as other industrial purposes (Alizadeh and Shahrodi n.d.).

## Conclusion

The results of this study showed that published articles in the field of nanotechnologies in the field of environmental health were few. Due to the positive impact of nanotechnology on environmental health in developed countries, it is recommended that scientists, professors, researchers and even students of environmental health in Iran as a developing country, expand their nanotechnology knowledge through interdisciplinary and regional and international partnerships. Reduce and eliminate environmental pollutants such as water, air and food pollution and other sub-sectors of environmental health.

The strengths of this study are that scientometric method and citation analysis were used to examine the status of scientific health products in the field of nanotechnology, which has been less addressed in environmental health issues. Analysis of a relatively wide range of time (for 11 years) is also one of the strengths of this study.

### Limitations

One of the limitations of this study is the lack of review of other environmental health articles in other medical journals.

## Data Availability

Not applicable.

## Abbreviations

US-EPA: United States Environmental Protection Agency
IJHE: Iranian Journal of Health and Environment
JEHE: Journal of Environmental Health Engineering
JREH: Journal of Research in Environmental Health
